# A Longitudinal Study on Early Hospitalized Airway Infections and Subsequent Childhood Asthma

**DOI:** 10.1371/journal.pone.0121906

**Published:** 2015-04-28

**Authors:** Mei-Jy Jeng, Yu-Sheng Lee, Pei-Chen Tsao, Chia-Feng Yang, Wen-Jue Soong

**Affiliations:** 1 Institute of Emergency and Critical Care Medicine, School of Medicine, National Yang-Ming University, Taipei, Taiwan, R.O.C; 2 Department of Pediatrics, Faculty of Medicine, School of Medicine, National Yang-Ming University, Taipei, Taiwan, R.O.C; 3 Department of Pediatrics, Taipei Veterans General Hospital, Taipei, Taiwan, R.O.C; Kliniken der Stadt Köln gGmbH, GERMANY

## Abstract

**Background:**

Acute airway infections, including bronchiolitis, are common causes of early childhood hospitalization. The development of later asthma may be related to early airway infections in young children. This study is to investigate the relationship between hospitalized airway infections (HAI) in young children (< 3 years old) and later childhood asthma.

**Methods:**

Hospitalized children (< 3 years old) with bronchiolitis or other acute airway infections (other HAI group) from 1997-2000 were retrieved from the National Health Insurance Research Database of Taiwan, and compared to age- and gender-matched subjects with regards to asthma until 10 years of age; and potential comorbidities and medical care conditions.

**Results:**

In total, 3,264 children (1,981 with bronchiolitis; 1,283 with other HAIs) were compared to 18,527 controls. The incidence of childhood asthma was higher in the study (16.2%) than the control (11.7%) group, and most cases were diagnosed between 3-5 years old. The hazard ratios were 1.583 (95% CI: 1.414-1.772) and 1.226 (95% CI: 1.053-1.428) for the bronchiolitis and other HAI subgroups, respectively, compared to the control group, and 1.228 (95% CI: 1.075-1.542) in the bronchiolitis subgroup compared to the other HAIs subgroup. A significantly higher odds ratio (1.973, 95% CI: 1.193-3.263) for the children with congenital heart disease (CHD) in the bronchiolitis subgroup was found at an age of 3-5 years compared to the control group.

**Conclusions and Clinical Relevance:**

Young children (< 3 years old) hospitalized due to acute HAIs are at a higher risk of developing childhood asthma at age 3 to 10 years. The parents of children with HAIs at age 0 to 2 years should be informed for the higher risk of developing childhood asthma, especially in children with CHD and bronchiolitis.

## Introduction

Acute airway infections are common causes of early childhood hospitalization. Among these infections, acute bronchiolitis is a viral infection commonly encountered in infants and toddlers with acute symptoms of wheezy cough and dyspnea [1, 2]. In addition, an increasing trend in medical visits and total hospitalizations along with the diagnosis of bronchiolitis has been reported in young children in recent decades [3, 4]. Asthma is a severe form of hyperactive airway disease which may present as an acute attack or chronic persistent pattern, and the influence on daily activities can be a serious problem. The clinical symptoms of wheezing and dyspnea in acute asthmatic attacks are similar to acute bronchiolitis. Therefore, whether there is a relationship between bronchiolitis and later childhood asthma, and even adulthood asthma has been investigated for many years [5–9]. It has been reported that recurrent wheezing, reduced pulmonary function, and the development of later asthma may occur in young children with bronchiolitis [10–23], however there is currently no conclusive evidence [24, 25].

In addition to bronchiolitis, other acute airway infections are not unusual in hospitalized infants and toddlers. Airway inflammation caused by infections may also be related to later childhood asthma. Elucidating the potential relationship between childhood asthma and various kinds of acute airway infections occurring in infants and toddlers is therefore warranted.

Taiwan′s National Health Insurance Research Database (NHIRD) includes comprehensive claims data from the National Health Insurance (NHI) program. This program covers more than 99.5% of residents in Taiwan, and children younger than 18 years of age account for approximately 23% of the whole population in Taiwan [26]. The NHIRD provides reliable data for population-based disease research [27–30]. These datasets contain aggregated secondary data without personal identification, including patient′s age, gender, admission date, discharge date, diagnosis, expenses, laboratory examination items, detail drug prescription codes and operational codes [31]. They have been used in extensive medical research fields from neonates to adults [26–28, 32–38]. It is trustworthy to analyze the population-based relationship between early acute airway infections and later childhood asthma development using these datasets.

We hypothesized that children with a history of early hospitalization due to acute airway infections may have a higher risk of childhood asthma. The purpose of this study was to analyze the relationship between early hospitalization due to acute airway infections, including acute bronchiolitis and other airway infections, in children younger than 3 years of age and subsequent childhood asthma when they are 3 to 10 years of age.

## Materials and Methods

### Study population

Claims data from the Longitudinal Health Insurance Database (LHID) 2010 of the National Health Insurance Research Database (NHIRD) of Taiwan between 1997 and 2010 were retrieved for analysis and comparisons. The LHID 2010 includes claims data of 1,000,000 randomly sampled beneficiaries from the 2010 registry of beneficiaries from the NHIRD. There are no significant differences in age distribution, gender distribution, or average insured payroll-related amount between the patients in the LHID 2010 and the original NHIRD according to the Bureau of National Health Insurance (NHI) in Taiwan [39, 40]. This study was approved by the Institutional Review Board of Taipei Veterans General Hospital, Taipei, Taiwan (VGHIRB No.:2013-06-011BC). There was no consent given because the data were analyzed anonymously and no personal information could be connected in this study.

**Fig 1:**
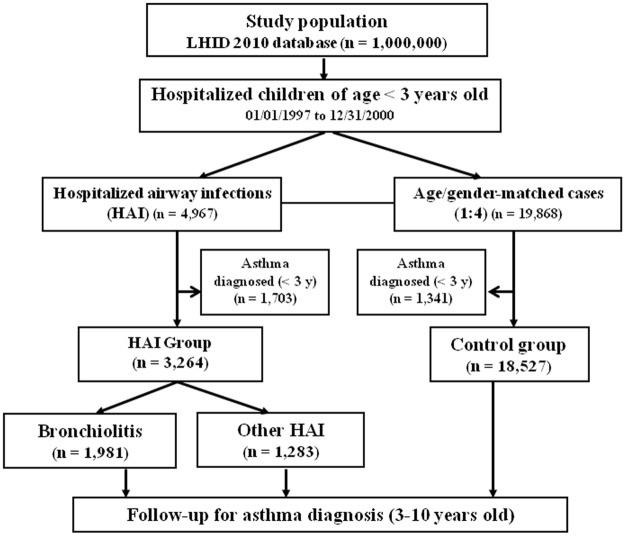
Flow chart of case enrollment from the National Health Insurance Research Database in Taiwan. LHID 2010, Longitudinal Health Insurance Database with random sampling of beneficiaries enrolled in 2010.

Information of children younger than 36 months who were hospitalized from 1997 to 2000 were retrieved, and those diagnosed with acute bronchiolitis and other acute airway infections on discharge were enrolled into our study group. The diagnostic codes were based on the International Classification of Diseases, 9th Revision, Clinical Modification (ICD-9-CM) codes, including acute bronchiolitis (466.1), and other acute airway infections (464.1–464.5, 466.0, and 480–486) (Table 1) [41]. The children in the control group were selected from the remaining patients during the same enrollment period who did not have any admission records or outpatient records of the above diagnoses. We randomly enrolled four times the number of age- and gender-matched children for the control group for comparisons. The exclusion criteria were a diagnostic record of asthma (ICD-9-CM: 493) at age younger than 3 years. The remaining children were grouped into hospitalized airway infection (HAI) and control groups, and the HAI group was further sub-grouped into bronchiolitis and other HAI subgroups (Fig 1). The basic data of the enrolled children, including age and gender were recorded and analyzed. Data on potential comorbidities including preterm (ICD-9-CM: 765), congenital heart disease (CHD) (ICD-9-CM: 745, 746, 747), congenital respiratory disease (ICD-9-CM: 748), and chronic lung disease (ICD-9-CM: 770.7) were also retrieved.

**Table 1:** Diagnostic Codes Defined as Acute Bronchiolitis, Other Acute Airway Infections and Asthma of the Enrolled Children. ^a^

ICD-9-CM Code	Diagnosis
**Acute bronchiolitis**	
**466.1**	Acute bronchiolitis
**Other acute airway infections**	
**464.1**	Acute tracheitis
**464.2**	Acute laryngotracheitis
**464.3**	Acute epiglottitis
**464.4**	Croup
**464.5**	Supraglottitis, unspecified
**466.0**	Acute bronchitis
**480**	Viral pneumonia
**481**	Pneumococcal pneumonia
**482**	Other bacteria pneumonia
**483**	Pneumonia-other specified organism
**484**	Pneumonia in infectious disease classified elsewhere
**485**	Bronchopneumonia, organism unspecified
**486**	Pneumonia, organism unspecified
**Asthma**	
**493**	Asthma

^a^Based on International Classification of Diseases, 9th Revision, Clinical Modification (ICD-9-CM) code.

Each child was then individually tracked by medical care records from 3 to 10 years of age. The record of a diagnosis of asthma (ICD-9-CM: 493) from the admission datasets (≥ 1 admission with discharge diagnosis of asthma) or outpatient datasets were recorded and analyzed. The diagnosis of asthma in the outpatient dataset was included only if they had 4 or more records of visits due to asthma, and having used one of the following anti-asthmatic medicines at each visit: adrenergics (Anatomical Therapeutic Chemical (ATC) codes: R03A, R03C), xanthines (R03DA), or steroids/β2 agonists (R03BA, R03AK06, R03AK07). The age at first diagnosis of asthma between 3 to 10 years was defined as the age at onset. Medical care conditions including ambulatory visit frequency, admission frequency, admission diagnosis, and medical expenses between 3 to 10 years of age were also retrieved and compared among groups.

### Data analysis

The dataset from the NHIRD was retrieved using Microsoft SQL Server 2008 R2 for database decoding, and SPSS (version 19, SPSS Inc., Chicago, IL, USA) was used for data analysis. SigmaPlot 12.0 (Systat Software Inc. San Jose, CA, USA) and SPSS were used to create graphical representations. One way ANOVA followed by post hoc Student Newman Keul or *t* tests were used to compare means of continuous variables as appropriate, and the chi-square test was used to compare categorical data among different groups. Logistic regression modeling was used to analyze odds ratios (ORs) of the study group compared to the control group. Kaplan-Meier survival analysis with the log rank test, Breslow test, and Tarone-Ware test was used to compare the cumulative asthma event-free ratios of the enrolled children among different groups. For time-to-event analysis of the longitudinal follow-up, Cox regression analysis was performed to analyze hazard ratios between any two groups. A two-sided *p* value of less than 0.05 was considered to determine statistical significance.

## Results

A total of 4,967 young children hospitalized with acute airway infections were identified, and we identified another 19,868 age- and gender-matched children for comparison. After excluding those with an early (< 36 months of age) diagnosis of asthma, there were 3,264 children in the HAI group, including 1,981 children with bronchiolitis and 1,283 children with other HAIs, and 18,527 children in the control group (Fig 1). There were more boys than girls in each group. The mean age of the children at first diagnosis of bronchiolitis was significant younger than that in the other HAI subgroup (*p* < 0.001) and the mean index age of control group at enrollment (*p* < 0.001) (Table 2).

**Table 2:** Characteristics of the children at enrollment and onset of childhood asthma at age 3 to 10 years.

	HAI group			Control group
	Total	Bronchiolitis	Other HAI	
**At enrollment (0–2 years old)**				
Case no.	3264	1981	1283	18527
Age (months, mean ± SD)	12.9 ± 9.6	9.5 ± 7.7^a^ ^*c*^	18.2 ± 9.9^a^	12.5 ± 9.4
Male (% of total)	1891 (57.9)	1184 (59.8)	707 (55.1)^b^	11026 (59.5)
Female (% of total)	1373 (42.1)	797 (40.2)	576 (44.9)^b^	7501 (40.5)
**Onset of childhood asthma (3–10 years old)**				
Case number				
3–10 y (% of enrolled case)*	530 (16.2)^a^	351 (17.7)^a^	179 (14.0)^b^	2159 (11.7)
3–5 y (% of total)	411 (77.5)	272 (77.5)	139 (77.7)	1487 (68.9)
6–10 y (% of total)	119 (22.5)	79 (22.5)	40 (22.3)	672 (31.1)
Age				
(months, mean ± SD)	59.1 ± 19.2^a^	59.6 ±19.5^a^	58.2 ± 18.7^a^	64.8 ± 20.1
(years, mean ± SD)	4.9 ± 1.6^a^	5.0 ± 1.6^a^	4.8 ± 1.6^a^	5.4 ± 1.7
Gender				
Male (% of total)	322 (60.8)	219 (62.4)	103 (57.5)^b^	1404 (65.0)
Female (% of total)	208 (39.2)	132 (37.6)	76 (42.5)^b^	755 (35.0)

Abbreviations: HAI, hospitalized airway infection; y, years old.

*% of enrolled children with early childhood airway disease (0–2 years old).

^a^ *p* < 0.001 vs. control group;

^b^ *p* < 0.05 vs. control group,

^*c*^ *p* < 0.001 vs. other HAI subgroup.

With regards to the occurrence of asthma at 3 to 10 years old, there were 530 (16.2%) children diagnosed with asthma in the overall HAI group, which were significantly higher than the proportion of the control group (11.7%) (*p* < 0.05). The mean ages of the children diagnosed with asthma in the bronchiolitis and other HAI subgroups were both significantly younger than in the control group (*p* < 0.001). Although there were still more boys than girls in each group, the proportion of male children was significantly lower in other HAI subgroup (57.5%) than control group (65.0%) (Table 2).

**Fig 2:**
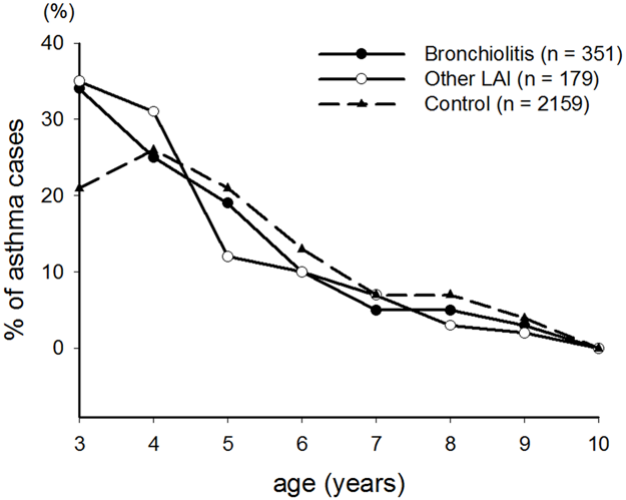
Age distribution of childhood asthma at 3–10 years in children with/without early hospitalized airway infections.

**Fig 3:**
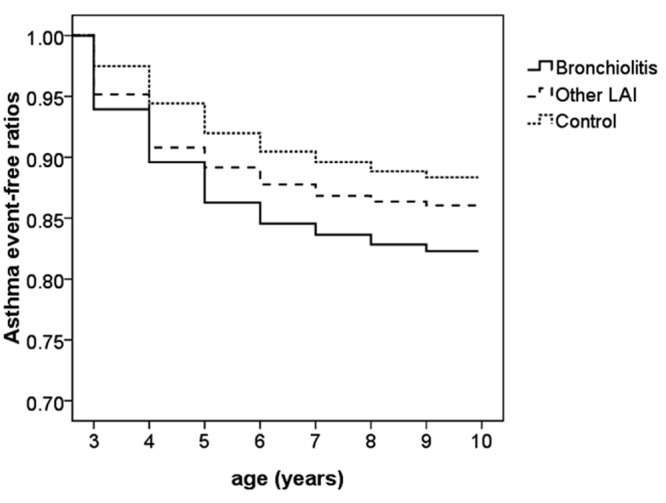
Cumulative asthma event-free rates of the enrolled children aged 3 to 10 years in different groups. Kaplan-Meier survival analysis was performed. *p* < 0.001 by the log rank test; *p* < 0.001 by the Breslow test; *p* < 0.001 by the Tarone-Ware test.

Most cases of asthma were diagnosed when they were 3 to 5 years old (approximately 78% in both HAI subgroups, and 69% in the control group) (Table 2), and the proportion of cases was markedly higher in both HAI subgroups than the control group at 3 years of age (Fig 2). The control group had the highest asthma event-free rate and the bronchiolitis subgroup had the lowest rate over time. There were significant differences in the asthma event-free rates during the 8 years of follow-up among the 3 groups (Fig 3) (*p* < 0.05). The time-to-event analysis showed that the hazard ratios were 1.583 (95% CI: 1.414–1.772), and 1.226 (95% CI: 1.053–1.428) in the bronchiolitis and other HAI subgroups compared to the control group, respectively. In addition, the hazard ratio of the bronchiolitis subgroup compared to the other HAI subgroup was 1.228 (95% CI: 1.075–1.542). Therefore, the risk of developing asthma at an age of 3–10 years was significantly higher in the bronchiolitis subgroup and the lowest in the control group, and the other HAI subgroup had a medium risk.

The ORs, adjusted for age and gender, of the children with early HAI for the occurrence of childhood asthma at an age of 3–10 years (*p* < 0.05) and of 3–5 years (*p* < 0.001) were significantly higher than in the control group for both bronchiolitis and other HAI subgroups (Table 3). Between the ages of 6–10 years, only the children with bronchiolitis still had a significantly higher OR compared to the control group (*p* = 0.018). For each potential comorbidity, the ORs of the study groups with regards to preterm, congenital respiratory disease, or chronic lung disease did not significantly differ from the control group (all *p* > 0.05). However, a significantly higher OR for the children with CHD in the bronchiolitis subgroup was noted at an age of 3–5 years compared to the control group (*p* = 0.008) (Table 3).

**Table 3:** Adjusted odds ratios of children with early hospitalized airway infections (HAIs) (< 3 years) and childhood asthma (3–10 years), and those with combined comorbidities of preterm birth, congenital heart disease, or congenital respiratory diseases compared to the control group.

		3–10 y	3–5 y	6–10 y
	Case no. (% of total)	aOR (95% CI)	aOR (95% CI)	aOR (95% CI)
**All children**				
HAI	3264 (100)	1.470 (1.328–1.630)	1.849 (1.624–2.106)	1.044 (0.894–1.218)
Bronchiolitis	1981 (100)	1.633 (1.443–1.848)	1.928 (1.648–2.256)	1.243 (1.038–1.487)
Other HAI	1283 (100)	1.229 (1.043–1.449)	1.730 (1.420–2.107)	0.745 (0.569–0.976)
Control	18527 (100)	1	1	1
**Preterm (ICD = 765)**				
HAI	110 (3.4)	1.164 (0.629–2.154)	1.557 (0.693–3.499)	0.824 (0.351–1.938)
Bronchiolitis	86 (4.3)	1.232 (0.639–2.376)	1.528 (0.641–3.645)	0.949 (0.389–2.315)
Other HAI	24 (1.9)	0.931 (0.296–2.929)	1.659 (0.436–6.311)	0.402 (0.051–3.179)
Control	164 (0.9)	1	1	1
**CHD (ICD = 745,746,747)**				
HAI	260 (8.0)	1.244 (0.856–1.808)	1.838 (1.160–2.911)	0.689 (0.382–1.243)
Bronchiolitis	187 (9.4)	1.458 (0.971–2.189)	1.973 (1.193–3.263)	0.911 (0.495–1.677)
Other HAI	73 (5.7)	0.754 (0.364–1.560)	1.503 (0.684–3.306)	0.156 (0.021–1.147)
Control	674 (3.6)	1	1	1
**CRD (ICD = 748)**				
HAI	47 (1.4)	0.656 (0.213–2.019)	1.310 (0.235–7.360)	0.452 (0.117–1.750)
Bronchiolitis	39 (2.0)	0.627 (0.194–2.028)	0.917 (0.142–5.925)	0.559 (0.143–2.179)
Other HAI	8 (0.6)	0.810 (0.130–5.028)	3.667 (0.424–31.726)	0
Control		1	1	1
**CLD (ICD = 770.7)**				
HAI	9 (0.0)	0.667 (0.059–7.475)	2.250 (0.125–40.656)	0
Bronchiolitis	8 (0.0)	0.762 (0.067–8.665)	2.571 (0.141–47.017)	0
Other HAI	1 (0.0)	0.0	0	0
Control	19 (0.0)	1	1	1

Abbreviations: y, years old; CHD, congenital heart disease; CLD, chronic lung disease arising in the perinatal period; CRD, congenital respiratory disease; HAI, hospitalized airway infections; aOR, adjusted odds ratio, adjusted by age and gender; ICD, International Classification of Diseases, 9th Revision, Clinical Modification code.

The analysis of subsequent medical care requirement of the enrolled children at an age of 3–10 years also showed significantly higher rates of total hospitalizations, ambulatory visits, and medical expenses for children with HAI, including both subgroups, compared to the control group (all *p* < 0.01) (Table 4). In each patient with asthma, the number of admissions with a diagnosis of asthma was significantly higher in the bronchiolitis subgroup (*p* < 0.001) than the control group, but not in the other HAI subgroup (*p* > 0.05) (Table 4). Comparing both HAI subgroups, the children in the bronchiolitis subgroup had a higher number of hospitalizations due to asthma, higher number of ambulatory visits, and overall medical expenses than the other HAI subgroup at age 3 to 10 years (all *p* < 0.01) (Table 4).

**Table 4:** Subsequent hospitalization and ambulatory visit frequencies and expenses in the enrolled children at age 3 to 10 years.

	HAI group*			Control group
	Total	Bronchiolitis	Other HAI	
**All hospitalizations (any diagnosis)**				
All enrolled case no.	3264	1981	1283	18527
Frequencies	2272	1525	747	6318
Hospitalization/case	0.7 ± 1.4^a^	0.8 ± 1.6 ^a^	0.6 ± 1.1^a^	0.3 ± 0.9
**Hospitalizations due to asthma**				
Asthma case no.	530	351	179	2159
Frequencies	726	559	167	1729
Hospitalizations/asthma case	1.4 ± 2.1^a^	1.6 ± 2.4^a^ ^b^	0.9 ± 1.3	0.8 ± 1.8
**Frequency of ambulatory visits**				
Frequencies	420,226	264,764	155,462	2,119,346
Visits/child	129 ± 76^a^	134 ± 77^a^ ^b^	121 ± 74^a^	114 ± 67
**Medical expense**				
All expense (USD$)	5,734,192	3,211,446	37,261,008	37,261,008
Expense/case (USD$)	2,741 ± 7,066^a^	2,895 ± 8,854^a^ ^b^	2,503 ± 4,843^a^	2,011 ± 2,538

Abbreviations: HAI, hospitalized airway infections; USD, United States dollar.

*children who were admitted due to lower airway infections when they were younger than 3 years old.

^a^ *p* < 0.01 vs. control group.

^b^ *p* < 0.01 vs. other HAI group.

## Discussion

The results of this study showed a significantly higher occurrence of childhood asthma in children who were hospitalized at an early age (< 3 years old) due to acute airway infections, including both bronchiolitis and other acute HAIs. Furthermore, we also demonstrated that most cases of asthma developed in children aged 3–5 years, the presence of CHD increased the risk in children with bronchiolitis, and the risk of early bronchiolitis was higher than other HAIs.

Viruses are the leading causes of acute lower respiratory tract infections in young children, and respiratory syncytial virus (RSV) has been reported to be the most common pathogen [1, 42]. Previous investigations have mostly focused on the role of RSV infection-associated bronchiolitis and the development of later childhood asthma [10, 12–20, 25], however some studies have also reported the influence of non-RSV viruses [23, 43–46]. The current study investigated the potential influence of relatively severe acute airway infections in children requiring hospitalization regardless of the causal organisms, and we found that acute airway infections other than bronchiolitis also increased the risk of subsequent childhood asthma. This suggests that attention should be paid to young children hospitalized for different types of acute airway infections due to the possibility of later childhood asthma.

Previous investigations on the epidemiology of bronchiolitis in Taiwan have demonstrated that bronchiolitis in young children occurs all year round with two slight peaks in spring and autumn [11, 47–49], and this is different from North America and Europe where a predominant peak is noted in winter [1, 2, 23]. Ethnic and environmental differences may exist, however published reports tend to show a higher likelihood of developing asthma or recurrent wheezing in children who suffer from early bronchiolitis in many countries [11, 12, 14, 21, 23, 45, 50, 51]. Therefore, the risk of developing asthma in children with early airway infections seems a general phenomenon, and our results also support this phenomenon in children living in Taiwan.

With regards to the age at onset, our results showed that most cases of childhood asthma were initially diagnosed when the children were 3–5 years old, regardless of whether their initial problems were bronchiolitis, other HAIs, or neither of these (Table 3). In particular, there were significantly more cases of asthma in both HAI groups than in the control group at 3 years of age (Fig 2). This means that children with early HAIs are more likely to suffer from an earlier onset of childhood asthma.

Sigurs et al. reported that the development of asthma, recurrent wheezing or impaired pulmonary function is more common in children with RSV-infected bronchiolitis [13–16]. However, some meta-analyses have reported no significant differences compared to control groups in children older than 5 years [52–54]. In the current study, we only analyzed the development of childhood asthma. Although the ORs of the bronchiolitis subgroup at an age of 6–10 years were still significant (*p* = 0.018), they were not as high as in those between 3–5 years (Table 3). In addition, in the other HAI group, the risk of asthma was not higher than the control group at an age of 6–10 years. These findings suggest that infection-associated airway hyperactivity may gradually resolve as the children grow older and their airways become larger.

Prematurity, CHD, and chronic lung disease have been reported to be potential risk factors for severe bronchiolitis in early childhood [55]. In children born prematurely, the risk of developing childhood asthma was reported to be higher than full-term children [56, 57]. Our study demonstrated that early HAI would not further increase the risk of developing asthma in preterm-born children, nor would the children with CRD or CLD. In children with CHD, the relationship to childhood asthma was rarely reported [58]. Our results revealed that the occurrence of bronchiolitis in children with CHD significantly increased the risk of developing asthma at age 3–5 years. Therefore, children with CHD and who suffer from bronchiolitis should be followed carefully for the potential risk of developing later childhood asthma, and their parents should be informed about this.

Genetic factors have been reported to be associated with early bronchiolitis and later asthma in children [59, 60]. In the current study, we focused on the association between severe airway infections and later asthma in children living in Taiwan. However, we lacked data to compare genetic differences, and further investigations are warranted to elucidate this issue.

Comparing the bronchiolitis and other HAI subgroups at an age of 6–10 years, we demonstrated higher odds ratios, more medical care requirement, and a higher number of hospitalizations due to asthma in the children with bronchiolitis (Tables 3 and 4). These findings suggest that bronchiolitis may have a more severe influence on airways than other HAIs, although both conditions can increase the risk of later asthma development at an age of 3–5 years. Thus, we suggest pediatricians to pay more attention to children suffering from bronchiolitis for the development of later childhood asthma up to 10 years of age.

Considering the cause and effect relationship between early HAIs and later childhood asthma, there may be some possibilities. For one thing, children with asthmatic characteristics may have susceptible airways and be easier to get HAIs during early childhood. For another thing, early HAIs in little kids may influence their airway development, and further increase the risk of developing asthma during later childhood. These two mechanisms may be synergistic. The present study is only an observation using claims data, so it is not easy to elucidate the cause-effect relationship. A further study on this issue will be necessary in the future.

There are some limitations to the present study. The main limitation is that NHIRD is derived from claims data of medical care providers in the NHI program, but the laboratory data and patients′ history were not included. Therefore, accurate viral or bacterial diagnoses could not be obtained, and we could not further evaluate the influence of microbial patterns in childhood asthma. We also could not evaluate the role of other potential confounding factors, such as patients′ birth weight, presence of older siblings, maternal smoking, family circumstances, etc. In addition, the diagnosis of asthma was based on the diagnostic codes of the in- and out-patients claims data without supporting evidence of pulmonary function tests.

## Conclusions

Young children hospitalized with bronchiolitis or other HAIs during the first 3 years of life may have a higher risk of developing childhood asthma at age 3 years or older, and require more medical care and expense in the following years. The parents of children with HAIs at age 0–2 years should be informed that there is a higher incidence of childhood asthma and that they should see a physician upon signs of obstructive airway disease, especially in children with bronchiolitis and underlying CHD, and at an age of 3 to 5 years.
